# Resource Provisioning in Fog Computing: From Theory to Practice †

**DOI:** 10.3390/s19102238

**Published:** 2019-05-14

**Authors:** José Santos, Tim Wauters, Bruno Volckaert, Filip De Turck

**Affiliations:** Department of Information Technology, Ghent University—imec, IDLab, Technologiepark-Zwijnaarde 126, 9052 Gent, Belgium; Tim.Wauters@UGent.be (T.W.); Bruno.Volckaert@UGent.be (B.V.); Filip.DeTurck@UGent.be (F.D.T.)

**Keywords:** smart cities, IoT, fog computing, resource provisioning, Kubernetes

## Abstract

The Internet-of-Things (IoT) and Smart Cities continue to expand at enormous rates. Centralized Cloud architectures cannot sustain the requirements imposed by IoT services. Enormous traffic demands and low latency constraints are among the strictest requirements, making cloud solutions impractical. As an answer, Fog Computing has been introduced to tackle this trend. However, only theoretical foundations have been established and the acceptance of its concepts is still in its early stages. Intelligent allocation decisions would provide proper resource provisioning in Fog environments. In this article, a Fog architecture based on Kubernetes, an open source container orchestration platform, is proposed to solve this challenge. Additionally, a network-aware scheduling approach for container-based applications in Smart City deployments has been implemented as an extension to the default scheduling mechanism available in Kubernetes. Last but not least, an optimization formulation for the IoT service problem has been validated as a container-based application in Kubernetes showing the full applicability of theoretical approaches in practical service deployments. Evaluations have been performed to compare the proposed approaches with the Kubernetes standard scheduling feature. Results show that the proposed approaches achieve reductions of 70% in terms of network latency when compared to the default scheduling mechanism.

## 1. Introduction

In recent years, the Internet-of-Things (IoT) rapidly started gaining popularity due to the wide adoption of virtualization and cloud technologies. IoT services have been introducing a whole new set of challenges by transforming everyday life objects into smart connected devices [1]. With the advent of IoT, Smart Cities [2] have become an even more attractive business opportunity. Smart Cities aim to reshape different domains of urban life, such as waste management, public transportation and street lightning. According to [3], by 2022, nearly three-quarters of all connected devices in the mobile network are expected to be smart devices. Additionally, the share of Low-Power Wide-Area Network (LPWAN) connections is expected to grow from about 2 percent in 2017 to 14 percent by 2022, from 130 million devices in 2017 to 1.8 billion devices by 2022. LPWANs are low-power wireless connectivity solutions specifically meant for Machine-to-Machine (M2M) use cases requiring wide geographic coverage and low bandwidth. Nowadays, the centralized structure of cloud computing is facing tremendous scalability challenges to meet the decentralized nature of IoT services due to the enormous bandwidth demands, high mobility coverage and low latency requirements [4]. As an answer, Fog Computing [5,6] has emerged as an extension to the Cloud Computing paradigm by distributing resources on the edges of the network close to end devices, thus, helping to meet the demanding constraints introduced by IoT services. Waste management platforms, Augmented Reality applications, video streaming services and smart transportation systems are already envisioned Smart City use cases for Fog Computing, which will benefit from the nearby real-time processing and data storage operations to overcome the limitations of traditional cloud architectures [7]. Although the theoretical foundations of Fog Computing have already been established, the adoption of its concepts is in early stages. Practical implementations of Fog Computing solutions are scarce. Additionally, research challenges in terms of resource provisioning and service scheduling still persist. In fact, setting up a proper Fog-based architecture to support millions of devices and their high demand heterogeneous applications without dismissing the importance of network latency, bandwidth usage and geographic coverage is still a challenge to be addressed in Fog Computing [8].

Nowadays, container-based micro-services are revolutionizing software development [9]. Micro-services represent an architectural style inspired by service-oriented computing that has recently started gaining popularity. An application is decomposed in a set of lightweight autonomous containers deployed across a large number of servers instead of the traditional single monolithic application [10]. Each micro-service is developed and deployed separately, without compromising the application life-cycle. Currently, containers are the *de facto* alternative to the conventional Virtual Machine (VM), due to their high and rapid scalability and their low resource consumption. In fact, due to their broad acceptance, several research projects are being conducted on container technologies by IT companies and open-source communities. The most popular among them is called Kubernetes [11]. Kubernetes is an open-source container management platform originally developed by Google. Kubernetes simplifies the deployment of reliable, scalable distributed systems by managing the complete orchestration life-cycle of containerized applications. Although containers already provide a high level of abstraction, they still need to be properly managed, specifically in terms of resource consumption, load balancing and server distribution, and this is where integrated solutions like Kubernetes come into their own [12]. Therefore, in this article, a Fog Computing architecture based on the Kubernetes platform for Smart City deployments is presented. The proposed architecture has been designed for Antwerp’s City of Things testbed [13]. Furthermore, intelligent allocation decisions are crucial for proper resource provisioning in Fog environments. Multiple factors should be taken into account, such as response time, energy consumption, network latency, reliability, bandwidth usage and mobility [14]. Although Kubernetes already provides provisioning functionalities, the scheduling feature merely takes into account the number of requested resources (CPU, RAM) on each host, which is rather limited when dealing with IoT services.

Thus, a network-aware scheduling approach presented in [15] has been implemented as an extension to the default scheduling feature available in Kubernetes to enable resource allocation decisions based on the current status of the network infrastructure. Last but not least, an Integer Linear programming (ILP) formulation for the IoT service placement problem presented in [16] has been deployed on the Kubernetes container orchestration platform, showing the full applicability of theoretical approaches in practical service deployments. Finally, evaluations based on Smart City container-based applications have been performed to compare the performance of the proposed provisioning mechanisms with the standard scheduling feature present in Kubernetes.

The remainder of the article is organized as follows. In the next Section, related work is discussed. Then, in Section 3, the importance of proper resource provisioning in Fog Computing is highlighted. Section 4 introduces the proposed Fog-based Kubernetes architecture for the resource provisioning of container-based services in Smart City deployments and its scheduling features. In Section 5, the proposed scheduling extensions in Kubernetes are discussed. Then, in Section 6, the evaluation setup is described which is followed by the evaluation results in Section 7. Finally, conclusions are presented in Section 8.

## 2. Related Work

In recent years, several studies have been carried out to deal with resource provisioning issues in Smart City deployments specifically tailored to IoT services. In [17], a reference Fog-based architecture has been presented. Their approach focused on implementing a Software Defined Resource Management layer at the Fog layer to locally serve IoT requests. Among different functionalities, a resource provisioning module has been included which is responsible for making allocation decisions based on metrics gathered by a monitoring module. In [18] both architectural and resource allocation concepts have been tackled. The authors proposed a provisioning algorithm focused on service elasticity and on the number of available resources by using virtualization technologies. Simulation results have shown that the proposed algorithm efficiently schedules resources while minimizing the response time and maximizing the throughput, without any consideration to the overall cost. Furthermore, in [19], a resource scheduling approach based on demand predictions has been presented. Their work focuses on allocating resources based on users’ demand fluctuations by using cost functions, different types of services and pricing models for new and existing customers. The model achieves a fair performance by preallocating resources based on user behavior and future usage predictions.

Additionally, in [20], the IoT resource provisioning issue has been modeled as an optimization problem. The model considered the maximization of Fog resources and the minimization of overall network delay. Their work has been extended in [21], where application Quality of Service (QoS) metrics and deadlines for the provisioning of each type of application have been taken into account. In [22], a hybrid approach for service orchestration in Fog environments is introduced. The solution encompasses two stages. On one hand, at the IoT and South-Bound Fog Levels, distributed management is proposed, which applies choreography techniques to enable automated fast decision making. On the other hand, centralized orchestration is suggested at the North-Bound Fog and Cloud Levels. In [23], an algorithm for workload management in Fog infrastructures has been presented. Their work focuses on task distribution at the Fog layer while minimizing response time based on resources demanded by these tasks. However, specific QoS requirements have not been considered in their approach. In [24], a service provisioning approach for combined fog-cloud architectures has been formulated as an optimization problem. Their model focuses on the minimization of network latency while guaranteeing proper service operation. Furthermore, in [25], an optimization formulation for the service deployment of IoT applications in Fog scenarios has been proposed and implemented as a prototype called FogTorch. Their work focused not only on hardware and software demands but also on QoS requirements, such as network latency and bandwidth.

In summary, this work advances beyond existing and ongoing studies that individually address some of the remaining challenges, but have not yet delivered an autonomous and complete solution for proper resource provisioning in Fog Computing. In this article, a Fog-based Kubernetes architecture is proposed to enable the deployment of Smart City container-based services, while increasing the performance over existing network infrastructure to fully maximize the potential of new business opportunities triggered by IoT and Smart City use cases. It combines Fog Computing concepts alongside the flexible and powerful Kubernetes platform to improve the performance of application-to-resource provisioning schemes. By combining powerful container orchestration technologies as Kubernetes and Fog Computing concepts, the proposed approach paves the way towards a proper resource provisioning in the Smart City ecosystem.

## 3. Open Challenge: Resource Provisioning in Fog Computing

This section highlights the importance of proper resource provisioning in Fog environments.

### Relevance of Proper Resource Provisioning

Resource provisioning is related to the allocation of computing, network and storage resources needed to instantiate and deploy applications and services requested by clients and devices over the Internet. Fog Computing has been introduced to address the inherent challenges of computing resource allocation for IoT services in Smart City deployments. Services can be provisioned in a highly congested location, or even further from sensors, which would result in a higher communication latency since current sensors and gateways are lacking in terms of processing power, storage capacity and memory [26]. Centralized solutions are not suitable for IoT since sending all the collected data to the cloud is unfeasible due to the high bandwidth requests. Fog Computing provides data processing and analytics operations locally, which drastically reduces the amount of data needed to transport to the cloud [27]. Furthermore, appropriate responses to protect infrastructure components as well as application level communication can be executed in a timely manner if malfunctions or abnormal events are detected in the data.

**Figure 1:**
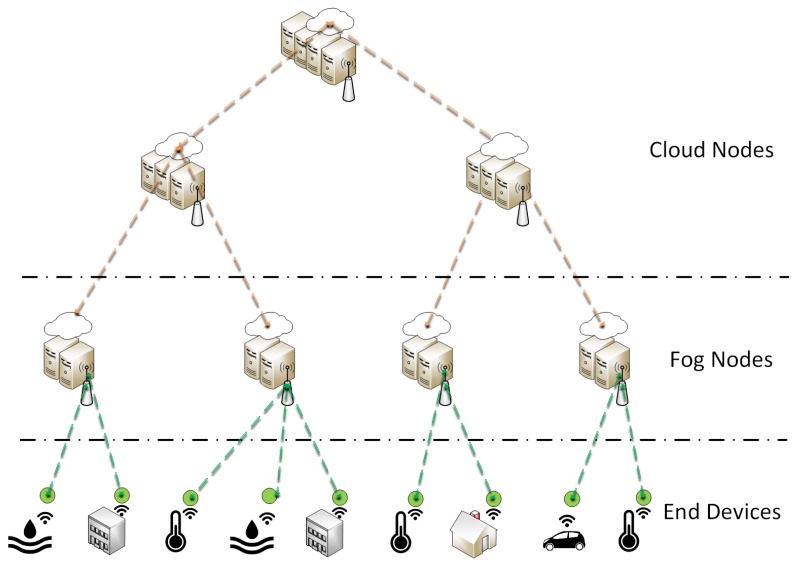
High-level view of Fog Computing.

Figure 1 presents a high-level view of the Fog Computing architecture. Opposed to a centralized cloud solution, end devices, sensors and actuators mainly communicate through wireless gateways, which are linked with a Fog layer through multiple Fog Nodes (FNs). The communication with the Cloud layer is then performed through Cloud Nodes (CNs). Nevertheless, as previously mentioned, concrete implementations of Fog Computing concepts are still in early stages and several issues still remain unresolved in resource provisioning for Fog Computing architectures:

- **Latency:** IoT services are highly challenging in terms of latency demands, since delay-sensitive applications, such as connected vehicles and health-care monitoring services, require low latencies in the order of milliseconds. If the latency threshold is exceeded, the service can become unstable, action commands may arrive too late and control over the service is potentially lost. Fog Computing is essential to provide low latencies to these delay-sensitive services.
- **Bandwidth:** The available bandwidth between sensors and the cloud is a serious constraint in Smart Cities. Datasets are so huge that the amount of bandwidth needed to transport all the data to the cloud is unacceptable. For instance, considering a video surveillance use case, where a video camera requires a connection of 10 Mb/s. Continuously sending the data from the video camera to the cloud translates into approximately 3.24 TB/monthly for a single camera. It is therefore essential to adopt Fog Computing concepts to perform data analysis operations locally, thus, reducing the amount of data transferred to the cloud.
- **Energy efficiency:** IoT devices are usually resource-constrained regarding their battery capacity, computational power, size and weight. For instance, considering a smart lightning use case, where thousands of sensors are measuring and controlling the light intensity of street lampposts. These sensors periodically wake up, measure values, send data samples to the network and then enter a sleep mode. Then, FNs perform the required computational operations on behalf of the sensors on the data collected to ensure an extension of the devices’ lifetime.
- **Programmability:** Fog Computing solutions are currently being designed as software driven technologies [28]. A Fog service provider will own a set of distributed Fog and Cloud Nodes where all hierarchical levels are simple to access and the whole software stack is easy to setup and maintain. Thus, the economic value of IoT is in the application software and the infrastructure running it. In fact, software modules are needed for life-cycle management and orchestration of Smart City services, including resource provisioning mechanisms.
- **Reliability:** Emergency and fire rescue services have extremely demanding availability requirements. In case of malfunctions or failures on a given FN, nearby FNs must be able to allocate the necessary resources to keep the provisioned Smart City services running properly. The hierarchical nature of Fog Computing architectures can improve the networks’ reliability by allowing distributed and local decision making in terms of resource provisioning.
- **Mobility:** Several IoT use cases have demanding mobility requirements. For instance, consider a connected waste management fleet, where trucks are continuously moving through the City. Messages must be sent to trucks alerting for possible accidents or roadblocks that may occur on their predefined route. However, due to interference, network overload or even dead coverage spots the connectivity between the FN and the truck may be lost. Therefore, FNs must work together to find the best solution for the allocation of each service instance being requested by a moving device to ensure adequate service operation at all times. High mobility services require the deployment of Fog Computing technologies since centralized management approaches cannot fully satisfy the dynamic demands of these type of services. Thus, Fog Computing is essential to rapidly modify the allocation of services according to highly variable demand patterns.
- **Decentralized Management:** The available computing resources must be distributed towards the edge of the network closer to end devices and users [29]. The so-called FNs provide local operations towards improving the response time in terms of resource allocation by efficiently scheduling all the necessary operations in the provisioning workflow. FNs should be aware of the network status and possible anomalies and malfunctions that may occur to react accordingly and keep all the services operating properly. Fog Computing brings intelligence and processing power close to end devices, which increases the networks’ robustness and reliability.
- **Scalability:** Fog Computing has to accommodate different IoT use cases and must possess adequate capacity to deal with growing service demands. IoT services must run without disruption and support millions of devices. Fog Computing architectures must be designed with scalability constraints in mind. FNs require modular software platforms where updates and modifications can be easily made without service interruptions. As network demands increase, FNs can receive additional software modules providing functionalities to deal with the growing service usage.

## 4. Fog-Based Kubernetes Architecture for Smart City Deployments

This section introduces a Fog Computing architecture based on the Kubernetes platform. First, a system overview of the proposed architecture is detailed, followed by the presentation of its main concepts. Then, the scheduling feature of Kubernetes is discussed.

### 4.1. Kubernetes: Empowering Self-Driving Orchestration of Smart City Container-Based Applications

**Figure 2:**
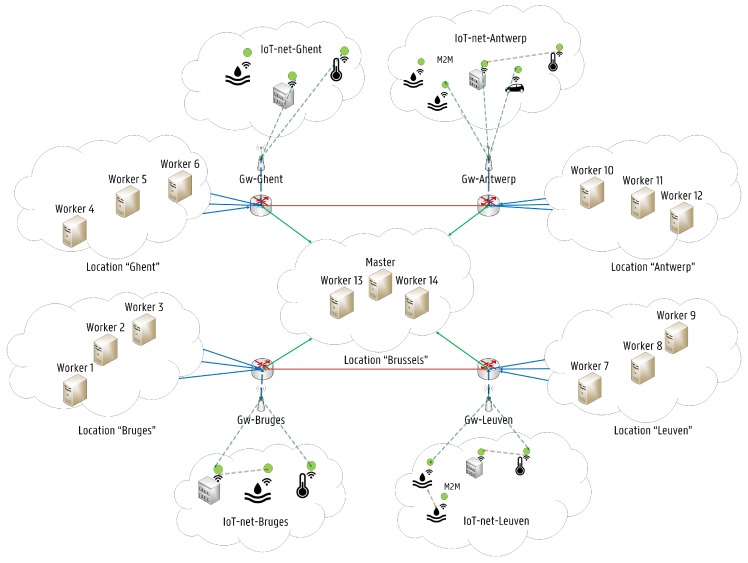
High-level view of the proposed Fog Computing infrastructure based on the Kubernetes platform.

**Figure 3:**
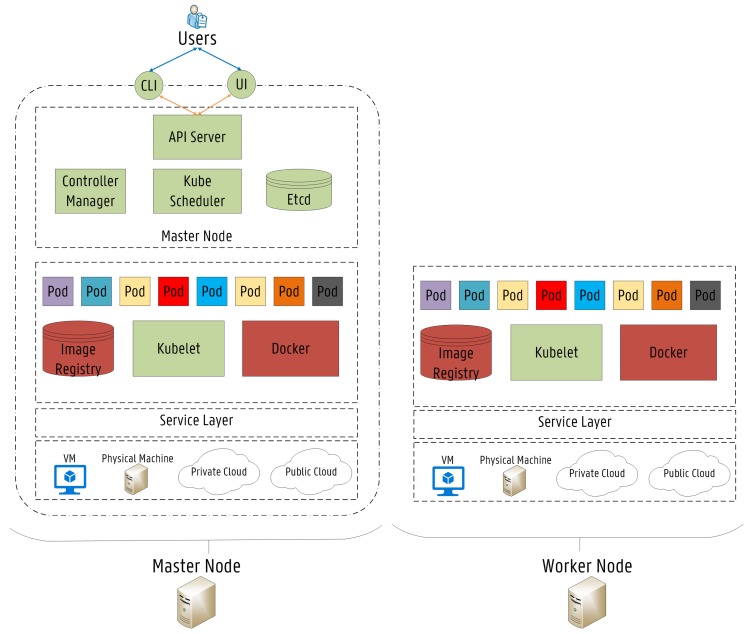
Detailed Architecture of the Master and the Worker Node in the Kubernetes Cluster [15].

The concept of Self-driving Orchestration has been introduced in [30] where it has been used to describe networks capable to measure, analyze and control themselves in an automated manner when reacting to changes in their environment. Kubernetes open source community is working towards a complete self-driving platform, aiming to simplify management and orchestration of scalable distributed systems across a wide range of environments and cloud providers for containerized applications. Kubernetes already provides orchestration features, which can be used to build reliable distributed systems with a high degree of decentralization in terms of the service life-cycle management, which is needed to fully leverage on Fog Computing architectures [31]. The proposed Fog-based Kubernetes architecture is shown in Figure 2. Several IoT networks are connected through wireless gateways to each of the represented locations. The architecture follows the master-slave model, where at least one master node manages Docker [32] containers across multiple worker nodes (slaves). End devices such as sensors are considered neither as master nor worker nodes. The proposed architecture follows the FN approach, where each FN is considered as a small cloud entity. The detailed architecture of the master and the slave nodes is shown in Figure 3. Nodes can be local physical servers and VMs or even public and private clouds. The Master is responsible for exposing the Application Program Interface (API) server, the scheduling of service deployments and managing the overall cluster life-cycle. Users interact with Kubernetes by communicating with the API server, which provides an entry point to control the entire cluster. Users can also send commands to the API server through the built-in Kubernetes Command Line Interface (CLI), known as Kubectl or even by accessing a web-based Kubernetes User Interface (UI). Another fundamental component is Etcd. Etcd is a lightweight key-value pair distributed data storage. Namespaces, scheduled jobs, deployed micro-services are examples of data stored in Etcd allowing other components to synchronize themselves based on the desired state of the cluster [33]. Furthermore, the main contact point for each cluster node is a service called Kubelet. Kubelet is responsible for recognizing discrepancies between the desired state and the actual state of the cluster. When this happens, Kubelet launches or terminates the necessary containers to reach the desired state described by the API server. Then, the Controller Manager is responsible for monitoring Etcd and the overall state of the cluster. If the state changes, the desired modifications are communicated through the API server. The Controller Manager is also responsible for the overall control of the runtime environment, including the creation and termination of containers.

Although Kubernetes makes use of containers as the underlying mechanism to deploy micro-services, additional layers of abstraction exist over the container runtime environment to enable scalable life-cycle orchestration features. In Kubernetes, micro-services are often tightly coupled together forming a group of containers. This is the smallest working unit in Kubernetes, which is called a pod [12]. A pod represents the collection of containers and storage (volumes) running in the same execution environment. The containers inside a pod share the same IP Address, volumes and port space (namespace), while containers in different pods are isolated from one another, since they own different IP addresses, different hostnames, etc. The main limitation is that two services listening on the same port cannot be deployed inside the same pod. Based on the available resources, the component that actually assigns pods to specific nodes in the cluster is called Kube–Scheduler (KS). The KS is the default scheduling feature in the Kubernetes platform, which is responsible for monitoring the available resources in the infrastructure and deciding on which adequate nodes pods should be placed. The selected node then pulls the required container images from the Image Registry and coordinates the necessary operations to launch the pod. The KS mechanisms are further detailed in the next section.

### 4.2. Resource Scheduling in Kubernetes: The Kube–Scheduler (KS)

**Figure 4:**
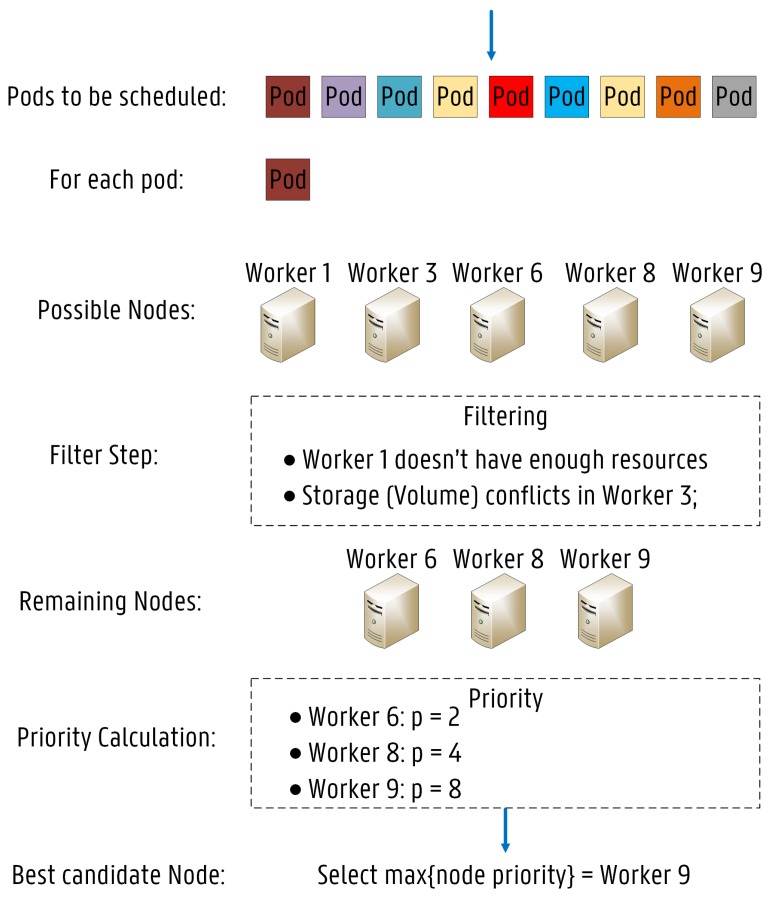
Sample of detailed scheduling operations of the Kube–Scheduler.

The KS decision making process is illustrated in Figure 4. Every pod requiring allocation is added to a waiting queue, which is continuously monitored by the KS. If a pod is added to the waiting queue, the KS searches for an adequate node for the provisioning based on a two step procedure. The first step is called node filtering, where the KS verifies which nodes are capable of running the pod by applying a set of filters, also known as predicates. The purpose of filtering is to solely consider nodes meeting all specific pod requirements further in the scheduling process. The second operation is named node priority calculation, where the KS ranks each remaining node to find the best fit for the pod provisioning based on one or more scheduling algorithms, also called priorities. The KS supports the following predicates [15,33]:

- **Check Node Memory Pressure:** This predicate checks if a pod can be allocated on a node reporting memory pressure condition. Currently, Best Effort pods should not be placed on nodes under memory pressure, since they are automatically deassigned from the node.
- **Check Node Disk Pressure:** This predicate evaluates if a pod can be scheduled on a node reporting disk pressure condition. Pods can currently not be deployed on nodes under disk pressure, since they are automatically deassigned.
- **Host Name:** This predicate filters out all nodes, except the one specified in the Spec’s NodeName field of the pod configuration file.
- **Match Node Selector (Affinity/Anti-Affinity):** By using node selectors (labels), it is possible to define that a given pod can only run on a particular set of nodes with an exact label value (node-affinity), or even that a pod should avoid being allocated on a node that has already certain pods deployed (pod-anti-affinity). These rules can be created by declaring Tolerations in the pod configuration files to match specific node Taints. Essentially, affinity rules are properties of pods that attract them to a set of nodes or pods, while taints allow nodes to repel a given set of pods. Taints and tolerations ensure that pods are not deployed onto inappropriate nodes. Both are important mechanisms to fine-tune the scheduling behavior of Kubernetes. Node selectors provide a flexible set of rules, on which the KS bases its scheduling decision by filtering specific nodes (node affinity/anti-affinity), by preferring to deploy certain pods close or even far away from other pods (pod affinity/anti-affinity), or just on node labels favored by the pod (taints and  tolerations).
- **No Disk Conflict:** This predicate evaluates if a pod can fit due to the storage (volume) it requests, and those that are already mounted.
- **No Volume Zone Conflict:** This predicate checks if the volumes a pod requests are available through a given node due to possible zone restrictions.
- **Pod Fits Host Ports:** For instance, if the pod requires to bind to the host port 80, but another pod is already using that port on the node, this node will not be a possible candidate to run the pod and, therefore, it will be disqualified.
- **Pod Fits Resources:** If the free amount of resources (CPU and memory) on a given node is smaller than the one required by the pod, the node must not be further considered in the scheduling process. Therefore, the node is disqualified.

The KS knows in advance which nodes are not suitable for the pod deployment by applying these predicates. Inadequate nodes are removed from the list of possible candidates. On one hand, after completion of the filtering process, finding no capable nodes for the pod deployment is always a possibility. In that case, the pod remains unscheduled and the KS triggers an event stating the reason for the failed deployment. On the other hand, if several candidates are retrieved after completion of the filtering operation, the KS triggers the node priority calculation. The node priority calculation is based on a set of priorities, where each remaining node is given a score between 0 and 10, 10 representing “perfect fit” and 0 meaning “worst fit”. Then, each priority is weighted by a positive number, depending on the importance of each algorithm, and the final score of each node is calculated by adding up all the weighted scores [33]. The highest scoring node is selected to run the pod. If more than one node is classified as the highest scoring node, then one of them is randomly chosen. The KS supports the following priorities [15]:

- **Balanced Resource Allocation:** This priority function ranks nodes based on the cluster CPU and Memory usage rate. The purpose is to balance the resource allocation after the pod provisioning.
- **Calculate AntiAffinity Priority:** This priority function scores nodes based on anti-affinity rules. For instance, spreading pods in the cluster by reducing the same number of pods belonging to the same service on nodes with a particular label.
- **Inter Pod Affinity Priority:** This priority algorithm ranks nodes based on pod affinity rules. For example, nodes with certain pods already allocated are scored higher, since it is preferred to deploy the given pod close to these pods.
- **Image Locality Priority:** Remaining nodes are ranked according to the location of the requested pod container images. Nodes already having the requested containers installed are scored higher.
- **Least Requested Priority:** The node is ranked according to the fraction of CPU and memory (free/allocated). The node with the highest free fraction is the most preferred for the deployment. This priority function spreads the pods across the cluster based on resource consumption.
- **Most Requested Priority:** This priority algorithm is the opposite of the one above. The node with the highest allocated fraction of CPU and memory is the most preferred for the deployment.
- **Node Affinity Priority:** In this case, nodes are scored according to node-affinity rules. For instance, nodes with a certain label are ranked higher than others.
- **Selector Spread Priority:** This priority algorithm tries to minimize the number of deployed pods belonging to the same service on the same node or on the same zone/rack.
- **Taint Toleration Priority:** This priority function scores nodes based on their taints and the correspondent tolerations declared in the pod configuration file. Remaining nodes are preferred according to the number of intolerable taints on them for the given pod. An intolerable taint is specified by the “Prefer No Schedule” key.

Predicates are evaluated to dismiss nodes that are incapable of running the given pod while priorities are designed to score all the remaining nodes that can deploy the pod. For example, a given node would be scored lower for the Selector Spread Priority if an instance of the requested pod is already allocated on that node. However, if a pod affinity rule is specified in the pod configuration file for the service, the node would be scored higher for the Inter Pod Affinity Priority since it is preferred to deploy the given pods close to each other. Furthermore, if a given pod requires a core CPU (1.0), the Pod Fits Resources predicate returns “False” for a node that only has 800 millicpu free. Additionally, for the same pod, the Most Requested Priority ranks a node that has only 200 millicpu free higher than one with 3.5 cores CPU left, even though both nodes can accommodate the pod (assuming they have the same CPU capacity). It should be noted that the KS searches for a suitable node for each pod, one at a time. The KS does not take the remaining pods waiting for deployment into account in the scheduling process. When the allocation decision is made, the KS informs the API server indicating where the pod must be scheduled. This operation is called Binding.

Another aspect worth mentioning of the Kubernetes provisioning life-cycle is called resource requests and limits. Developers can specify resource requests and limits on the pod configuration files. A resource request is the minimum amount of resources (CPU and/or memory) required by all containers in the pod while a resource limit is the maximum amount of resources that can be allocated for the containers in a pod. Pods can be categorized in three QoS classes depending on resource requests and limits:

- **Best Effort (lowest priority):** A Best Effort pod has neither resource requests or limits on its configuration files for each of its containers. These pods are the first ones to be terminated in case the system runs out of memory.
- **Burstable:** A Burstable pod has all containers with resource requests lower than their resource limits. If a container needs more resources than the ones requested, the container can use them as long as they are free.
- **Guaranteed (highest priority):** A guaranteed pod has resource requests for all its containers equal to the maximum resource needs that the system will allow the container to use (resource limit).

If resource requests are specified, the KS can provide better allocation decisions. Similarly, if resource limits are described, resource contention can be handled properly [34]. When several containers are running on the same node, they compete for the available resources. Since container abstraction provides less isolation than VMs, sharing physical resources might lead to a performance degradation called resource contention. Resource requests and limits enable Kubernetes to properly manage the allocation of resources. Nevertheless, developers still need to accurately set up these requests and limitations, because containers often do not use the entire amount of resources requested which could lead to wasted resources. For example, two pods have been deployed and each one is requesting 4 Gb of RAM in a node with 8GB RAM capacity, but each pod is only using 1 GB of RAM. The KS could allocate more pods onto that node, however, due to the incorrect specification in terms of resource requests, the KS will never schedule additional pods onto that node.

## 5. Resource Scheduling Extension in Kubernetes

This section introduces the proposed extensions to the default scheduling mechanism available in Kubernetes. First, a network-aware scheduling approach is detailed. Then, the ILP formulation implemented as a container-based application is discussed.

### 5.1. Network-Aware Scheduler (NAS) Implementation in Kubernetes

Although the KS provides flexible and powerful features, the metrics applied in the decision making process are rather limited. Only CPU and RAM usage rates are considered in the service scheduling while latency or bandwidth usage rates are not considered at all. A suitable scheduling approach for Fog Computing environments must consider multiple factors, such as the applications’ specific requirements (CPU, memory, minimum bandwidth), the state of the infrastructure (hardware and software), the network status (link bandwidth and latency), among others. Therefore, this article presents a Network-Aware Scheduler (NAS) extension to Kubernetes, which enables Kubernetes to make scheduling decisions based on up-to-date information about the current status of the network infrastructure. Kubernetes describes three ways of extending the KS:

- Adding new predicates and/or priorities to the KS and recompiling it.
- Implementing a specific scheduler process that can run instead of or alongside the KS.
- Implementing a “scheduler extender” process that the default KS calls out as a final step when making scheduling decisions.

**Figure 5:**
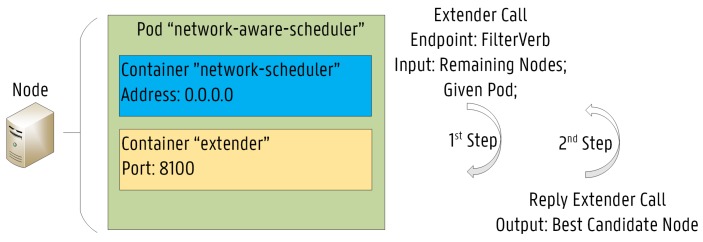
The detailed Pod architecture of the Network-Aware Scheduler (NAS).

**Figure 6:**
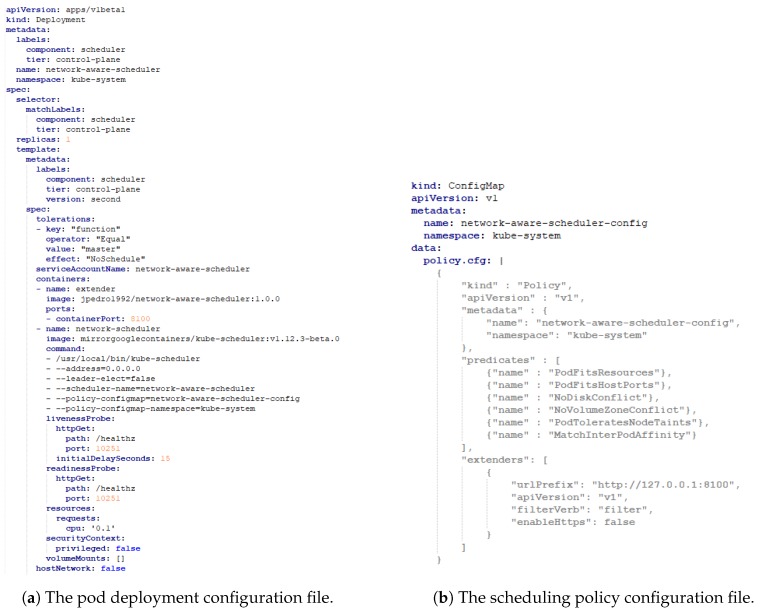
The configuration files required for the NAS.

The third approach is particularly suitable for use cases where scheduling decisions need to be made on resources not directly managed by the standard KS. The proposed NAS has been implemented based on this third approach, since information on the current status of the network infrastructure is not available throughout the scheduling process of the KS. The proposed NAS has been implemented in Go and deployed in the Kubernetes cluster as a pod. The pod architecture of the NAS is illustrated in Figure 5. Additionally, the pod configuration file for the NAS is shown in Figure 6a, while the scheduling policy configuration file for the NAS is presented in Figure 6b. As shown, the pod is composed of two containers: the extender and the NAS. The extender is responsible for performing the proposed scheduling operation, while the NAS is in fact the actual KS. A specific scheduler policy configuration file has to be defined to instruct the KS how to reach the extender and which predicates should be used to filter the nodes as a first step in the scheduling process. Essentially, when the KS tries to schedule a pod, the extender call allows an external process to filter the remaining nodes (second step). The arguments passed on to the “Filter Verb” endpoint consists of the set of nodes filtered through the KS predicates and the given pod. This second step is used to further refine the list of possible nodes.

A complete labeling of the Fog Computing infrastructure previously shown has been conducted based on Affinity/Anti-Affinity rules and node labels mentioned in Section 4.2. As illustrated, the infrastructure is composed of a Kubernetes cluster with 15 nodes (1 master node and 14 worker nodes). Nodes have been classified with labels “Min, Med, High” for keywords “CPU, RAM”, depending on their resource capacity. Additionally, nodes have been classified in terms of device type, by classifying them with taints “Cloud, Fog” for the keyword “Device Type” and according to their geographic distribution. Round Trip Time (RTT) values have been assigned to each node as a label so that delay constraints can be considered in the scheduling process. The labels of each node are listed in Table 1. These labels enable the placement of services in specific zones or certain nodes based on the location delay. All these rules are important to fine-tune the scheduling behavior of Kubernetes, in particular, to help the scheduler make more informed decisions at the filtering step by removing inappropriate  nodes.

The proposed NAS makes use of these strategically placed RTT labels to decide where it is suitable to deploy a specific service based on the target location specified in the pod configuration file. In fact, the node selection is based on the minimization of the RTT depending on the target location for the service after the completion of the filtering step. Additionally, in terms of bandwidth, NAS checks if the best candidate node has enough bandwidth to support the given service based on the pod bandwidth requirement. If the bandwidth request is not specified in the pod configuration file, a default value of 250 Kbit/s is considered during the scheduling phase. After completion of the scheduling request, the available bandwidth is updated on the corresponding node label. The NAS Algorithm is shown in Algorithm 1.

In summary, the proposed NAS approach filters the infrastructure nodes based on KS predicates and then makes use of the implemented RTT location labels to choose the best candidate node from the filtered ones to the desired service location.

**Algorithm 1** NAS Algorithm**Input: Remaining Nodes after Filtering Process** in **Output: Node for the service placement** out  1://Handle a provisioning request 2:handler(http.Request){ 3: receivedNodes=decode(http.Request); 4: receivedPod=decodePod(http.Request); 5: node=selectNode(receivedNodes,receivedPod); 6: **return** node 7:} 8:  9://Return the best candidate Node (recursive)10:selectNode(receivedNodes,receivedPod){11: targetLocation=getLocation(receivedPod);12: minBandwidth=getBandwidth(receivedPod);13: min=math.MaxFloat64;14: copyReceivedNodes=receivedNodes;15: 16: // find min RTT17: **for** node in range receivedNodes{18:  rtt=getRTT(node,targetLocation);19:  min=math.Min(min,rtt);20: }21: 22: // find best Node based on RTT and minBandwidth23: **for** node in range receivedNodes{24:  **if** min==getRTT(node,targetLocation){25:   **if** minBandwidth≤getAvBandwidth(node){26:    **return** node;27:   }28:   **else**29:    copyReceivedNodes=removeNode(copyReceivedNodes,node);30:  }31: }32: 33: // Available min RTT Nodes are full in terms of Network Bandwidth!34: // Repeat the Process (Recursive)!35: // First: Check if copy is not empty36: **if** copyReceivedNodes==null37:  **return** null, Error(“No suitable nodes found!”);38: **else**39:  **return** selectNode(copyReceivedNodes,receivedPod);40:}

### 5.2. From Theory to Practice: ILP Model Implementation in Kubernetes as a Container-Based Application

**Figure 7:**
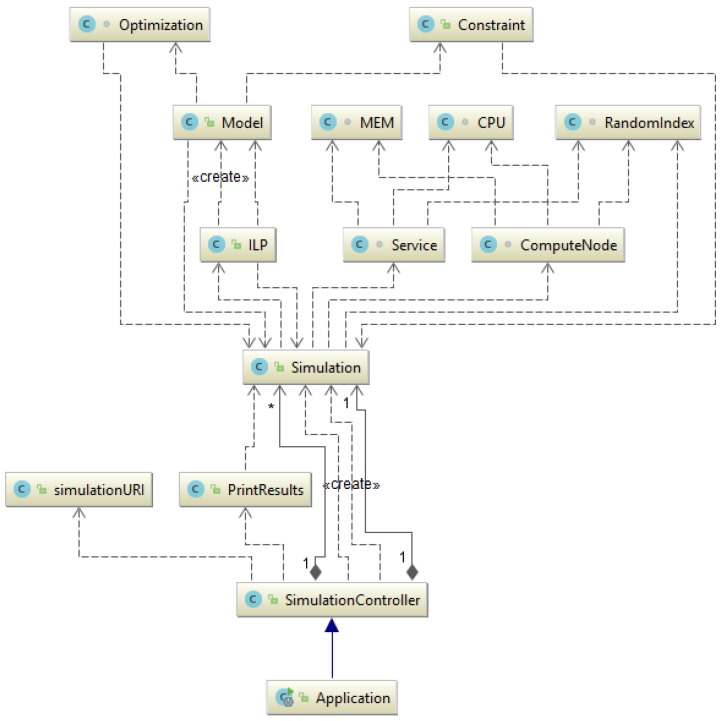
The class diagram of the ILP REST API generated with IntelliJ IDEA.

Lastly, an ILP model for the IoT service placement problem has been designed as a container-based application. The ILP model has been implemented in Java using the IBM ILOG CPLEX ILP solver [35] and the Spring Framework [36]. The class diagram of our implementation is shown in Figure 7. The class diagram has been generated with IntelliJ IDEA, a Java Integrated Development Environment (IDE) developed by Jetbrains [37]. The proposed ILP container application has been designed as a Representational State Transfer (REST) API. Simulation entities can be created or deleted through the Simulation Controller. ILP solutions can be obtained by issuing a GET Request for all Simulation entities available or by just sending a GET request for a specific Simulation entity. The ILP formulation incorporates multiple optimization objectives. Users can specify the desired optimization objectives and the amount of requests for each specific application when creating Simulation entities through a PUT request. In fact, the model is executed iteratively so that in each iteration a different optimization objective can be considered. To retain the solutions obtained in previous iterations, additional constraints are added to the model. Thus, the solution space continuously decreases since iterations must satisfy the previous optimal solutions. Every iteration refines the previously obtained solution by improving the model with an additional optimization objective.

**Figure 8:**
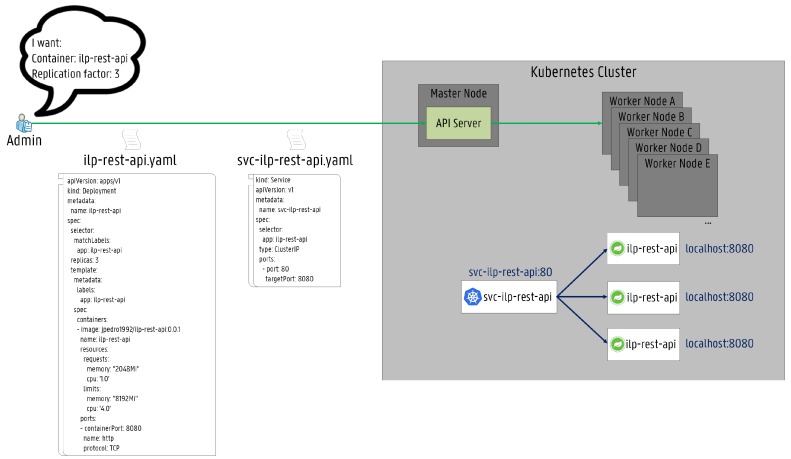
The detailed service scheme of the ILP REST API in the Kubernetes platform.

The main advantage of ILP is the flexibility to analyze complex problems such as the resource provisioning in Fog Computing presented in this paper. However, theoretical studies lack practical implementations, which limit their applicability to real deployments. Therefore, the proposed ILP REST API has been deployed and validated on the Kubernetes platform showing the full applicability of theoretical approaches in practical service deployments. In Figure 8, the proposed service architecture is shown. Two YAML Ain’t Markup Language (YAML) files are used to deploy the ILP REST API. Firstly, the ilp-rest-api.yaml is responsible for creating the deployment of the ILP REST API. The deployment is composed of three replicated ilp-rest-api Pods, indicated by the replicas field. This is the desired number of replicas. Additionally, for each pod, a core CPU (1.0) and 2 Gb of RAM are requested (resource requests), which can be increased to four cores and 8 Gb, respectively (resource limits). The service is listening on port 8080. Secondly, the svc-ilp-rest-api.yaml creates a Kubernetes Service called svc-ilp-rest-api. A Kubernetes Service is a flexible abstraction that provides a reliable manner to access a logical set of pods. The set of pods exposed are determined by a label selector, which in this case, corresponds to *“app: ilp-rest-api”*. Services make pods consistently accessible. Pods can be created, updated or even terminated so that the service will know exactly how many replicas are running, where pods are located and which IP addresses are being used. Essentially, services enable automatic load-balancing across several pods. The ClusterIP service type (default) has been used to provide internal access to the ilp-rest-api by exposing it on an internal IP in the cluster. Thus, the ilp-rest-api service is only reachable from within the cluster.

The proposed ILP REST API has been evaluated on the Kubernetes platform to compare the performance of the theoretical formulation with the implemented NAS approach and the standard scheduling feature available in Kubernetes. The evaluation use case is presented next.

## 6. Evaluation Use Case

In this section, the evaluation setup is detailed. Then, the evaluation scenario is described.

### 6.1. Evaluation Setup

**Figure 9:**
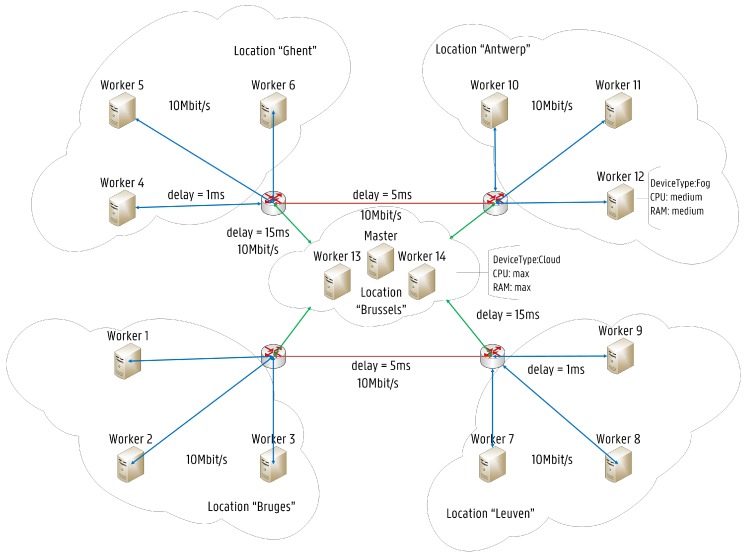
A Fog Computing infrastructure based on the Kubernetes platform.

The Kubernetes cluster has been set up on the imec Virtual Wall infrastructure [38] at IDLab, Belgium. The Fog Computing infrastructure illustrated in Figure 9 has been implemented with Kubeadm [39]. The cluster node hardware configurations are shown in Table 2. Furthermore, the software versions used to implement the Kubernetes cluster are listed in Table 3.

### 6.2. Scenario Description: Air Monitoring Service

**Figure 10:**
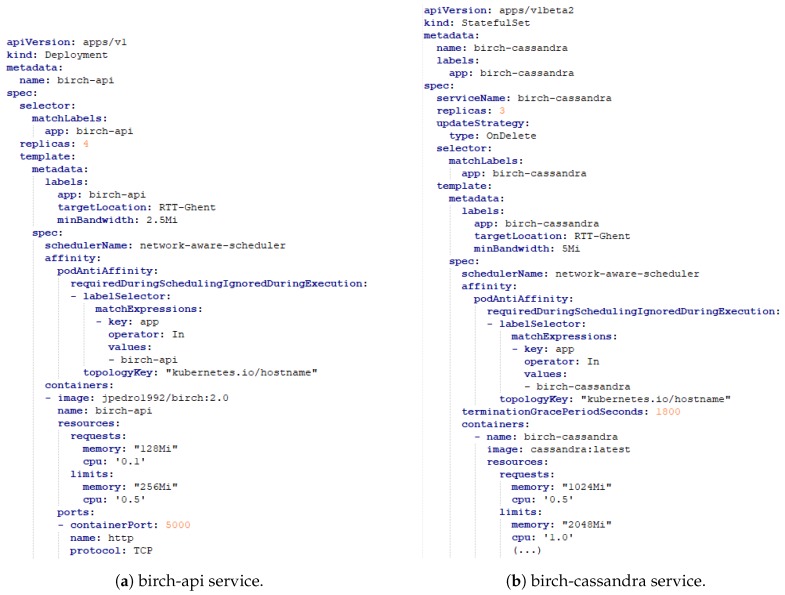
The pod configuration files for the Birch Service.

The evaluation use case is based on an Air Monitoring Service performing unsupervised anomaly detection. This scenario has been previously presented in [40], where a novel anomaly detection solution has been proposed for Smart City applications in Smart Cities based on the advantages of Fog Computing architectures. The purpose of this use case is to collect air quality data in the City of Antwerp to detect high amounts of organic compounds in the atmosphere based on outlier detection and clustering algorithms. Clustering allows the detection of patterns in unlabeled data while outlier detection is related to the identification of unusual data samples when compared to the rest of the dataset. In this article, the anomaly detection algorithms have been implemented as container APIs and then deployed as pods in the Kubernetes cluster. Regarding clustering, the Birch and the Kmeans algorithms have been evaluated while for outlier detection, the Robust Covariance and the Isolation Forrest have been assessed. The deployment properties of each service are shown in Table 4. In Figure 10, the pod configuration files for the deployment of the Birch service are presented. As shown, the service chain of the Birch service is composed of two pods, the API and the corresponding database. The desired location for the allocation of the service is expressed by the “targetLocation" label. Furthermore, the minimum required bandwidth per service is expressed by the “minBandwidth" label. As illustrated previously, the available bandwidth per node is 10.0 Mbit/s. Additionally, pod anti-affinity rules have been added to each service so that pods belonging to the same service chain are not deployed together, meaning that a node can only allocate one instance of a certain pod for a particular service. For instance, for the Birch service, the birch-api and the birch-cassandra pods cannot be deployed together. All pods have also been categorized as Burstable, since their containers have resource requests lower than their resource limits. The deployment of these services has been performed to compare the performance of our implemented approaches with the default KS.

### 6.3. ILP Model Configurations

In Table 5, the evaluated ILP model configurations are shown. First, for all model configurations, the number of accepted service requests is maximized in the first iteration. Then, on one hand, the ILP-A configuration corresponds to the minimization of service latency based on the service target location. On the other hand, the ILP-B configuration is related to the infrastructure’s energy efficiency, since the final goal of this configuration is to minimize the number of nodes used during the service provisioning. Finally, the ILP-C configuration corresponds to a joint optimization of latency and energy, where minimization of latency and the minimization of nodes correspond to the second and third iteration, respectively.

## 7. Evaluation Results

In this section, the evaluation results are detailed. First, the execution time of the different scheduling approaches is presented, followed by the correspondent scheduler resource consumption. Then, the allocation schemes for each of the schedulers is detailed. Finally, the average RTT per service and the expected service bandwidth per node for the different scheduling approaches are shown.

### 7.1. Scheduler Execution Time

In Table 6, the execution time of the different scheduler approaches is presented. Each evaluation run considered 24 pods as shown in Table 4 previously. The execution time has been evaluated 10 times. The scheduling decision of the default KS is made after on average 4.20 ms per pod, while the NAS requires on average 5.42 ms, due to the extender call procedure. The total execution time of the KS and the NAS is 126.08 ms and 162.74 ms, respectively. Additionally, the three ILP configurations previously presented have been requested to the ILP REST API. Firstly, the execution time of the ILP-A configuration is 1.82 s (first iteration: 0.86 s, second iteration: 0.96 s). Secondly, the execution time of the ILP-B configuration is 6.30 s (first iteration: 0.79 s, second iteration: 5.51 s). Thirdly, three objectives are considered in the ILP-C configuration. The ILP-C is a refined solution of the ILP-A configuration, since the minimization of the number of allocated nodes is considered as a third optimization objective, resulting in a higher execution time of 4.20 s (first iteration: 0.87 s, second iteration: 0.95 s, third iteration: 2.38 s). The higher execution time of the ILP-B configuration is due to the high potential solution space for minimizing energy in this scenario. Regarding the pod startup time, the KS and the NAS require on average 2 seconds to allocate and initialize the required containers while the ILP configurations need between 4 and 8 seconds due to the higher decision time. By comparing both the KS and the NAS with the three ILP formulations, it can be seen that heuristics can significantly reduce the execution time of ILP models.

### 7.2. Scheduler Resource Consumption

In Table 7, the resource consumption (CPU and RAM) of the different scheduler approaches is shown. As expected, the ILP REST API requires more resources than the other two scheduling mechanisms. Traditionally, ILP solvers need a high amount of resources and require high execution times to find optimal solutions to the given problem. Nevertheless, ILP techniques could improve the quality of the decision-making process by linearizing the problem and by only considering concrete objectives. The KS and the NAS have a similar resource consumption, since both schedulers are based on the default scheduling mechanism available in Kubernetes.

### 7.3. Allocation Scheme

**Figure 11:**
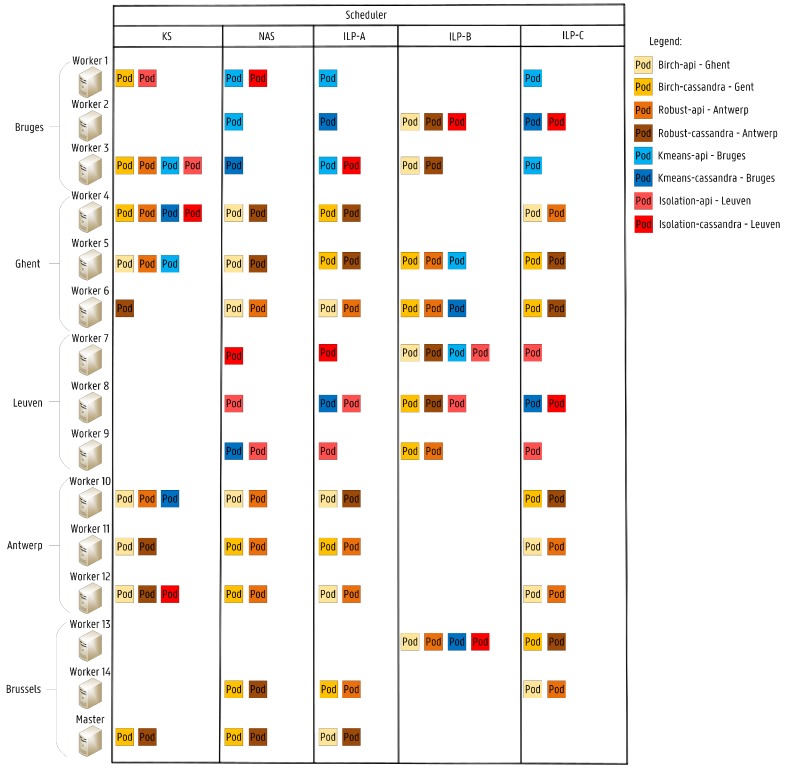
The service provisioning schemes of the different schedulers.

In Figure 11, the different allocation schemes for each of the schedulers are illustrated. As expected, the KS deployment scheme is not optimized for the service’s desired location, since no considerations are made about network latency in its scheduling algorithm. For instance, the KS allocation scheme of the Isolation service is fairly poor since both pods, isolation-api and isolation-cassandra, are not deployed in the desired location (Leuven). Furthermore, the ILP-B configuration is also not optimized for service latency, since the objective of the ILP is to minimize the energy consumption by just considering bandwidth constraints between services allocated on different nodes.

### 7.4. Network Latency and Bandwidth

**Figure 12:**
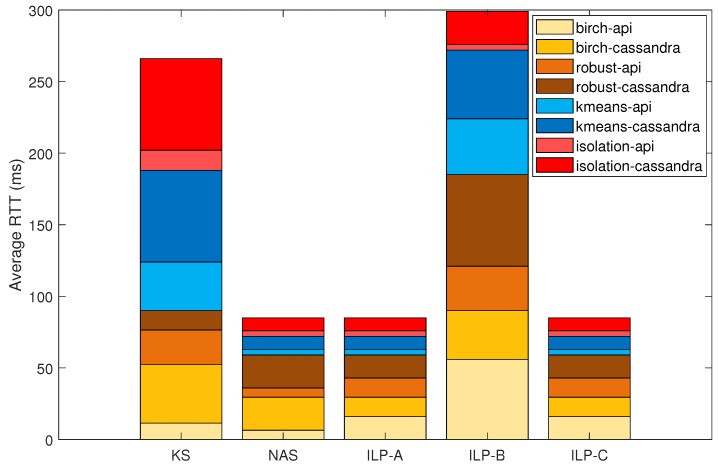
Comparison of the average RTT per scheduler for different pod-deployment scheduling strategies in a Smart City air quality monitoring scenario.

The differences in the average RTT per scheduler are detailed in Figure 12. As shown, the proposed NAS achieves significantly lower RTTs for each of the deployed services when compared with the default KS. Both NAS and ILP-A configuration achieve similar results in terms of the overall RTT. However, clear differences exist in the Birch and Robust service. RTT values of 6.5 ms and 23.0 ms are achieved with the NAS, while values of 16.0 ms and 13.5 ms are obtained with ILP-A. This difference occurs because the ILP takes all remaining pods waiting for deployment into account in the service provisioning, while the NAS searches for a suitable node for each pod, one at a time, similar to the KS. Therefore, the NAS optimizes first the birch-api and the robust-api services and just after their deployment, the correspondent birch-cassandra and robust-cassandra services are scheduled. The service provisioning in terms of network latency is highly improved with the NAS and the ILP-A configuration since KS and ILP-B do not consider bandwidth requests in the scheduling process. In this particular allocation scheme, the NAS improves the performance of the default KS by reducing the network latency by 70% while increasing the scheduling decision time by 1.22 ms per pod.

In Table 8, the expected service bandwidth per node for the different scheduling approaches is presented. Both KS and the ILP-B configuration allocate pods on nodes already compromised in terms of network bandwidth. For instance, KS overloads worker 4 and 12 by allocating to them at least 3 pods leading to service bandwidths of 17.0 Mbit/s and 12.5 Mbit/s for the workers 4 and 12, respectively, which surpasses the available bandwidth of 10.0 Mbit/s. This allocation scheme may lead to service disruptions due to bandwidth fluctuations. Furthermore, the ILP-B configuration overloads 5 worker nodes to reduce the number of active nodes to solely 8, meaning that the remaining 7 are not used in the service provisioning. This occurs due to the selected optimization objective (MIN Nodes). Additionally, it should be highlighted that, although ILP-A and ILP-C achieve the exact same values of RTT for each of the deployed services, their allocation scheme is quite different. ILP-C refines the solution obtained by ILP-A by trying to further optimize the solution space by considering the minimization of nodes as a third optimization objective while maintaining the same RTT values. As shown for this configuration, several nodes can be considered full in terms of network bandwidth since service bandwidths of 10 Mbit/s are expected, which is the limit in our network. Therefore, the ILP-C solution provides us a more efficient usage of the infrastructure by reducing the fraction of free resources per node.

In summary, the proposed NAS optimizes the resource provisioning in Kubernetes according to network latency and bandwidth, which is currently not supported by the default KS. An ILP REST API has been also validated as a container-based application to evaluate the performance of theoretical formulations in real service deployments. As shown, the execution time of ILP models is higher than heuristic mechanisms (KS and NAS). Nevertheless, ILP models obtain the optimal solution for the given problem based on a set of objectives. The evaluated ILP formulations improve the resource provisioning performance of the default KS in terms of latency or energy efficiency and even can refine the allocation scheme of the proposed NAS, while increasing the pod startup time on average by 4 seconds. It should be noted that a dynamic mechanism suitable for dealing with bandwidth fluctuations and delay changes is required, however, it is out of the scope of this article.

## 8. Conclusions

In this article, a Fog Computing architecture is proposed for the proper resource provisioning of Smart City container-based applications. Fog Computing has been introduced to manage the growing amount of connected devices in the upcoming years, by placing computational resources on the edges of the network. This trend has encouraged the development of scalable orchestration mechanisms to guarantee the smooth performance of IoT services. Fog Computing provides effective ways to overcome the high demanding requirements introduced by IoT use cases, such as low latency, high energy efficiency and high mobility. The popular open-source project Kubernetes has been used to validate the proposed solution. The scalable design of Kubernetes provides flexible abstractions between the micro-services and the underlying infrastructure. In this article, a network-aware scheduling approach is proposed, which enables allocation decisions based on the current status of the network infrastructure. Additionally, an ILP formulation for the IoT service placement problem has been designed as a container-based application and then validated on the Kubernetes platform showing the full applicability of theoretical approaches in real service deployments. Evaluations have been performed to compare the proposed scheduling mechanisms. Results show that the proposed NAS can significantly improve the service provisioning of the default KS by achieving a reduction of 70% in network latency, while increasing the scheduling decision time by only 1.22 ms per pod. Theoretical approaches can demonstrate their full applicability when applied to real service deployments as shown by the validated ILP REST API.

## Figures and Tables

**Table 1:** The implemented node labels in the Kubernetes cluster.

Node	Device Type	CPU	RAM	Bandwidth	RTT Ghent	RTT Antwerp	RTT Bruges	RTT Leuven	RTT Brussels
Master	Cloud	High	High	10.0 Mbit/s	32.0 ms	32.0 ms	32.0 ms	32.0 ms	4.0 ms
Worker 1	Fog	Min	Min	10.0 Mbit/s	64.0 ms	64.0 ms	4.0 ms	14.0 ms	32.0 ms
Worker 2	Fog	Med	Med	10.0 Mbit/s	64.0 ms	64.0 ms	4.0 ms	14.0 ms	32.0 ms
Worker 3	Fog	Min	Min	10.0 Mbit/s	64.0 ms	64.0 ms	4.0 ms	14.0 ms	32.0 ms
Worker 4	Fog	Min	Min	10.0 Mbit/s	4.0 ms	14.0 ms	64.0 ms	64.0 ms	32.0 ms
Worker 5	Fog	Med	Med	10.0 Mbit/s	4.0 ms	14.0 ms	64.0 ms	64.0 ms	32.0 ms
Worker 6	Fog	Med	Med	10.0 Mbit/s	4.0 ms	14.0 ms	64.0 ms	64.0 ms	32.0 ms
Worker 7	Fog	Min	Min	10.0 Mbit/s	64.0 ms	64.0 ms	14.0 ms	4.0 ms	32.0 ms
Worker 8	Fog	Med	Med	10.0 Mbit/s	64.0 ms	64.0 ms	14.0 ms	4.0 ms	32.0 ms
Worker 9	Fog	Min	Min	10.0 Mbit/s	64.0 ms	64.0 ms	14.0 ms	4.0 ms	32.0 ms
Worker 10	Fog	Med	Med	10.0 Mbit/s	14.0 ms	4.0 ms	64.0 ms	64.0 ms	32.0 ms
Worker 11	Fog	Med	Med	10.0 Mbit/s	14.0 ms	4.0 ms	64.0 ms	64.0 ms	32.0 ms
Worker 12	Fog	Min	Min	10.0 Mbit/s	14.0 ms	4.0 ms	64.0 ms	64.0 ms	32.0 ms
Worker 13	Cloud	Min	Min	10.0 Mbit/s	32.0 ms	32.0 ms	32.0 ms	32.0 ms	4.0 ms
Worker 14	Cloud	Med	Med	10.0 Mbit/s	32.0 ms	32.0 ms	32.0 ms	32.0 ms	4.0 ms

**Table 2:** The Hardware Configuration of each Cluster Node.

Node	CPU	RAM
Worker 1	2x Quad core Intel E5520 (2.2 GHz)	12 Gb
Worker 2	1x 4 core E3-1220v3 (3.1 GHz)	16 Gb
Worker 3	2x Quad core Intel E5520 (2.2 GHz)	12 Gb
Worker 4	2x Quad core Intel E5520 (2.2 GHz)	12 Gb
Worker 5	2x Hexacore Intel E5620 (2.4 GHz)	24 Gb
Worker 6	2x Hexacore Intel E5620 (2.4 GHz)	24 Gb
Worker 7	2x Dual core AMD opteron 2212 (2.0 GHz)	8 Gb
Worker 8	2x Hexacore Intel E5620 (2.4 GHz)	24 Gb
Worker 9	2x Dual core AMD opteron 2212 (2.0 GHz)	8Gb
Worker 10	2x Hexacore Intel E5620 (2.4 GHz)	24 Gb
Worker 11	2x Hexacore Intel E5620 (2.4 GHz)	24 Gb
Worker 12	2x Quad core Intel E5520 (2.2 GHz)	12 Gb
Worker 13	2x Dual core AMD opteron 2212 (2.0 GHz)	8Gb
Worker 14	2x Hexacore Intel E5620 (2.4 GHz)	24 Gb
Master	2x 8core Intel E5-2650v2 (2.6 GHz)	48 Gb

**Table 3:** Software Versions of the Evaluation Setup.

Software	Version
Kubeadm	v1.13.0
Kubectl	v1.13.0
Go	go1.11.2
Docker	docker://17.3.2
Linux Kernel	4.4.0-34-generic
Operating System	Ubuntu 16.04.1 LTS

**Table 4:** Deployment properties of each service.

Service Name	Pod Name	CPU Req/Lim (m)	RAM Req/Lim (Mi)	Min. Bandwidth (Mbit/s)	Rep. Factor	Target Location	Dependencies
Birch	birch-api	100/500	128/256	2.5	4	Ghent	birch-cassandra
	birch-cassandra	500/1000	1024/2048	5			birch-api
Robust	robust-api	200/500	256/512	2	4	Antwerp	robust-cassandra
	robust-cassandra	500/1000	1024/2048	5			robust-api
Kmeans	kmeans-api	100/500	128/256	2.5	2	Bruges	kmeans-cassandra
	kmeans-cassandra	500/1000	1024/2048	5			kmeans-api
Isolation	isolation-api	200/500	256/512	1	2	Leuven	isolation-cassandra
	isolation-cassandra	500/1000	1024/2048	5			isolation-api

**Table 5:** The evaluated ILP model configurations.

	ILP Configurations		
Iteration	ILP-A (Latency)	ILP-B (Energy)	ILP-C (Latency and Energy)
1st	MAX Requests	MAX Requests	MAX Requests
2nd	MIN Latency	MIN Nodes	MIN Latency
3rd	-	-	MIN NODES

**Table 6:** The execution time of the different schedulers.

Scheduler	Avg. Scheduling Decision (Per Pod)	Total Execution Time	Pod Startup Time
KS	4.20 ms	126.08 ms	2.04 s
NAS	5.42 ms	162.74 ms	2.13 s
ILP-A	-	1.82 s	3.97 s
ILP-B	-	6.30 s	8.45 s
ILP-C	-	4.20 s	6.35 s

**Table 7:** The resource consumption of the different schedulers.

Scheduler	Used CPU (m)	Used RAM (Mi)
KS	102	41.93
NAS	102	56.67
ILP-A	233	452.36
ILP-B	639	630.06
ILP-C	438	636.71

**Table 8:** The expected service bandwidth per node for the different scheduling strategies.

	Schedulers				
Node	KS	NAS	ILP-A	ILP-B	ILP-C
Worker 1	6.0 Mbit/s	7.5 Mbit/s	2.5 Mbit/s	-	2.5 Mbit/s
Worker 2	-	2.5 Mbit/s	5.0 Mbit/s	**12.5 Mbit/s**	10.0 Mbit/s
Worker 3	**10.5 Mbit/s**	5.0 Mbit/s	7.5 Mbit/s	7.5 Mbit/s	2.5 Mbit/s
Worker 4	**17.0 Mbit/s**	7.5 Mbit/s	10.0 Mbit/s	-	4.5 Mbit/s
Worker 5	7.0 Mbit/s	7.5 Mbit/s	10.0 Mbit/s	9.5 Mbit/s	10.0 Mbit/s
Worker 6	5.0 Mbit/s	4.5 Mbit/s	4.5 Mbit/s	**12.0 Mbit/s**	10.0 Mbit/s
Worker 7	-	5.0 Mbit/s	5.0 Mbit/s	**10.5 Mbit/s**	1.0 Mbit/s
Worker 8	-	1.0 Mbit/s	6.0 Mbit/s	**11.0 Mbit/s**	10.0 Mbit/s
Worker 9	-	6.0 Mbit/s	1.0 Mbit/s	7.0 Mbit/s	1.0 Mbit/s
Worker 10	9.5 Mbit/s	4.5 Mbit/s	7.5 Mbit/s	-	10.0 Mbit/s
Worker 11	7.5 Mbit/s	7.0 Mbit/s	7.0 Mbit/s	-	4.5 Mbit/s
Worker 12	**12.5 Mbit/s**	7.0 Mbit/s	4.5 Mbit/s	-	4.5 Mbit/s
Worker 13	-	-	-	**14.5 Mbit/s**	10.0 Mbit/s
Worker 14	-	10.0 Mbit/s	7.0 Mbit/s	-	4.5 Mbit/s
Master	10.0 Mbit/s	10.0 Mbit/s	7.5 Mbit/s	-	-

## Abbreviations

The following abbreviations are used in this manuscript:

API	Application Program Interface
CLI	Command Line Interface
CN	Cloud Node
FN	Fog Node
IDE	Integrated Development Environment
ILP	Integer Linear Programming
IoT	Internet-of-Things
KS	Kube–Scheduler
LPWAN	Low Power Wide Area Network
M2M	Machine-to-Machine
NAS	Network-Aware Scheduler
QoS	Quality of Service
REST	Representational State Transfer
RTT	Round Trip Time
UI	User Interface
VM	Virtual Machine
YAML	YAML Ain’t Markup Language
